# Lung Perfusion Assessment by Bedside Electrical Impedance Tomography in Critically Ill Patients

**DOI:** 10.3389/fphys.2021.748724

**Published:** 2021-10-13

**Authors:** Mengru Xu, Huaiwu He, Yun Long

**Affiliations:** State Key Laboratory of Complex Severe and Rare Diseases, Department of Critical Care Medicine, Peking Union Medical College, Peking Union Medical College Hospital, Chinese Academy of Medical Sciences, Beijing, China

**Keywords:** lung perfusion, regional ventilation/perfusion match, electrical impedance tomography, pulmonary embolism, acute respiratory distress syndrome

## Abstract

As a portable, radiation-free imaging modality, electrical impedance tomography (EIT) technology has shown promise in the bedside visual assessment of lung perfusion distribution in critically ill patients. The two main methods of EIT for assessing lung perfusion are the pulsatility and conductivity contrast (saline) bolus method. Increasing attention is being paid to the saline bolus EIT method in the evaluation of regional pulmonary perfusion in clinical practice. This study seeks to provide an overview of experimental and clinical studies with the aim of clarifying the progress made in the use of the saline bolus EIT method. Animal studies revealed that the saline bolus EIT method presented good consistency with single-photon emission CT (SPECT) in the evaluation of lung regional perfusion changes in various pathological conditions. Moreover, the saline bolus EIT method has been applied to assess the lung perfusion in a pulmonary embolism and the effect of positive end-expiratory pressure (PEEP) on regional ventilation/perfusion ratio (V/Q) and acute respiratory distress syndrome (ARDS) in several clinical studies. The implementation of saline boluses, data analyses, precision, and cutoff values varied among different studies, and a consensus must be reached regarding the clinical application of the saline bolus EIT method. Further study is required to validate the impact of the described saline bolus EIT method on decision-making, therapeutic management, and outcomes in critically ill patients.

## Introduction

The evaluation of regional pulmonary perfusion is of great interest for cardiopulmonary management in the intensive care unit (ICU). For many years, researchers have been searching for imaging techniques suitable for the assessment of pulmonary blood flow. Recent years have witnessed substantial progress in this area, and several techniques for pulmonary perfusion assessment, including MRI, CT, PET, and single-photon emission CT (SPECT), have become available (Hopkins et al., 2012; McCollough et al., 2015; Bondesson et al., 2019; Kohli et al., 2019). However, these methods are rarely used in critically ill patients because of the non-bedside implementation, high risk of transfer, inconvenience, etc.

Electrical impedance tomography uses a single-ring external electrode to image changes in impedance within a transversal section of the lungs; it is a non-invasive, radiation-free imaging modality that enables the continuous bedside monitoring of ventilation. A multitude of studies has confirmed the feasibility and reliability of ventilation images obtained by electrical impedance tomography (EIT) to inform mechanical ventilation settings (PEEP titration, lung recruitment, etc.) in critically ill patients (Frerichs et al., 2017; Yoshida et al., 2019). Electrical impedance tomography is also able to assess lung perfusion distribution. There are two EIT methods used for assessing lung perfusion: the pulsatility and conductivity contrasting (saline) bolus methods. Recently, increasing attention has been given to the application of the saline bolus EIT method in the visual evaluation of regional pulmonary perfusion in severe patients. This study seeks to provide an overview of experimental and clinical studies with the aim of clarifying the progress made in the use of the saline bolus EIT method.

## Pulsatility-Based EIT for Assessing Lung Perfusion

The pulsatility-based EIT method of measuring cardiac-related impedance variation, which has been widely investigated in both unwell and healthy subjects (Frerichs et al., 2009; Grant et al., 2011; Li et al., 2014), features non-invasive and real-time perfusion monitoring. In the last two decades, developments in EIT signal separating techniques involving frequency-domain filtering (Frerichs et al., 2009), ECG gating (Vonk Noordegraaf et al., 1998), respiratory pause (Fagerberg et al., 2009), and principal component analysis (PCA) (Deibele et al., 2008) have optimized pulsatile signal processing. We summarized the clinical studies using pulsatility-based EIT assessments for pulmonary perfusion in healthy volunteers and patients with pulmonary hypertension (PH) (Table 1).

**Table 1:** Summary on pulsatility-based EIT in clinical studies.

**Researchers**	**Main research contents**	**Subjects**	**CRS separating method**	**Conclusion**
Vonk Noordegraaf et al. (1998)	To investigate the influence of several pulmonary haemodynamical parameters on the EIT signal, including stroke volume, large pulmonary artery distensibility, the extent of the pulmonary peripheral vascular bed and the origin of the diastolic wave.	Group A: 11 emphysematous patients and 9 healthy age-matched non-smoking control subjects; Group B: 14 healthy subjects; Group C: 3 cardiological patients with atrioventricular dissociation.	ECG-gating (20 cardiac cycles)	Cardiac related lung impedance changes were significantly smaller in emphysematous patients with a reduced peripheral pulmonary vascular bed, but were not related to stroke volume and distensibility of the right pulmonary artery.
Smit et al. (2002)	To examine the validity of EIT in the measurement of pulmonary vasodilatation in patients with when given the vasodilating agent epoprostenol.	8 patients diagnosed as primary or secondary PH and referred for reversibility testing with epoprostenol.	ECG-gating (100 cardiac cycles)	EIT is a reliable method to measure blood volume changes due to pharmacologically induced vasodilatation in the pulmonary bed.
Smit et al. (2003)	To examine the validity of EIT in the measurement of HPV and hypoxic pulmonary vasodilation in healthy volunteers and COPD patients.	Group 1: 7 healthy volunteers; Group 2: 6 clinically stable COPD patients.	ECG-gating (200 cardiac cycles)	EIT can detect blood volume changes due to HPV non-invasively in healthy subjects and hypoxic vasodilation in COPD patients.
Smit et al. (2006)	To evaluate the differences in the EIT signal of the pulmonary vascular bed between healthy subjects and patients with IPAH.	21 patients with IPAH and 30 healthy controls.	ECG-gating (100 cardiac cycles)	The impedance pulsation of the pulmonary vascular bed is reduced in IPAH in comparison with controls, indicating a reduced volume pulse.
Carlisle et al. (2010)	To describe the relative regional distribution in blood volume within the thorax, using EIT, in preterm infants receiving SIPPV+TTV and to compare this to the regional distribution of tidal ventilation.	26 infants <32 completed weeks' gestational age at birth and older than 24 h who were receiving SIPPV + TTV.	Frequency-domain-filtering	Lung perfusion may be distributed toward the non-dependent lung and that it differs from the distribution of ventilation. EIT is feasible, and well-tolerated, in preterm infants.
Grant et al. (2011)	To extend a previously reported frequency filtering technique to a spontaneously breathing cohort and assess the regional distributions of ventilation and perfusion and their relationship.	10 healthy adults (21–52 years old).	Frequency-domain-filtering	The modified filtration technique was demonstrated to be effective in separating the ventilation and perfusion signals in spontaneously breathing subjects. Gravity dependence was not seen in any perfusion distributions.
Proença et al. (2016)	The use of EIT for continuous monitoring of PAP	3 healthy male subjects.	ECG-gating	Results suggest the feasibility of non-invasive, unsupervised monitoring of PAP using pulsatile EIT.
Proença et al. (2020)	To evaluate if EIT could be used for the continuous, unsupervised and safe monitoring of PAP.	30 healthy volunteers (induced gradual increases in SPAP by exposure to normobaric hypoxemia).	ECG-gating	Results demonstrate the feasibility of accurately assessing changes in SPAP by EIT in healthy volunteers.
Hovnanian et al. (2021)	To assess the association between impedance variation of lung perfusion and hemodynamic profile, severity, and prognosis, suspected of PAH or worsening PAH patients were submitted simultaneously to RHC and EIT	35 patients composed the PAH group, and eight patients, the normopressoric group based on the results of the RHC.	ECG-gating (100 cardiac cycles)	Impedance change of lung perfusion is associated with hemodynamic status of PAH patients, with disease severity and survival, demonstrating EIT as a promising tool for monitoring patients with pulmonary vascular disease.

*EIT, Electrical impedance tomography; CRS, cardiac-related signals; HPV, hypoxic pulmonary vasoconstriction; PH, pulmonary hypertension; IPAH, idiopathic pulmonary arterial hypertension; SIPPV+TTV, synchronized, volume-targeted intermittent positive pressure ventilation; PAP, pulmonary artery pressure; SPAP, systolic PAP; PAH, pulmonary artery hypertension*.

The basic principle of the pulsatility method for estimating pulmonary perfusion is based on the measurement of pulsatile changes in pulmonary blood volume instead of real forward lung blood flow. Hence, many potential factors (such as changes in vascular tone, synchronous changes in air content, airway pressure, and distensibility of the small pulmonary vessels) could affect the accuracy of using cyclic pulsatile changes in regional impedance to reflect the proportion of lung blood flow in those regions. The validity of the pulsatility method for assessing perfusion has been questioned (Borges et al., 2012; Putensen et al., 2019). Studies have found that downstream pulmonary vascular resistance and the distensibility of small pulmonary vessels can impact EIT pulsatility data (Smit et al., 2004; Borges et al., 2012). Borges JB et al. found that pulsatile impedance increased within the collapsed lung region, which had reduced lung blood flow based on both the SPECT and saline bolus EIT methods (Borges et al., 2012). Although the pulsatility method could easily provide continuous information on lung perfusion, the conductivity contrast bolus injection method showed superiority in terms of feasibility and accuracy (Frerichs et al., 2002; Deibele et al., 2008; Borges et al., 2012).

## Principles of the Conductivity Contrast Bolus EIT Method

The conductivity contrast bolus method, also named contrast-enhanced EIT or the saline bolus EIT method, was performed by introducing a bolus of hypertonic saline into the pulmonary circulation during a respiratory hold. During the breath-hold, tidal impedance was lacking; however, chest impedance was relatively constant. Hence, the change in impedance caused by the saline bolus reflects forward lung blood flow. The high variation in the regional impedance indicates more saline passing through the corresponding region, which also indicates high local lung perfusion. According to the typical first-pass kinetics manifested in indicator-enhanced CT and MRI (Konstas et al., 2009; Ohno et al., 2016), hypertonic saline following the blood flow from the right atrium through the pulmonary circulation is indicative of a significant decline in electrical impedance due to its high conductivity. The impedance–time curves produced by saline boluses were reconstructed to fit a gamma variate model and were quantitively analyzed based on the Fick principle and the accumulated mass of the indicator in every region of interest (Mullani and Gould, 1983).

In general, there were two methods used to analyze the indicator dilution, namely, the transfer model and the slope analysis. The slope analysis was validated against PET in animal studies (Borges et al., 2012; Bluth et al., 2019). Several recent publications on lung perfusion using EIT and saline bolus injections in human subjects adopted the slope analysis (He et al., 2020a,b; Mauri et al., 2020; Spinelli et al., 2021). A high slope in the lung regional time–impedance curves obtained following a saline injection indicates a high accumulation of saline, which also reflects a high lung blood perfusion.

More recently, Kircher et al. optimized an EIT-gamma function fitting based on a linear piecewise approximation of the drift model in a porcine acute respiratory distress syndrome (ARDS) model, simplifying the quantization algorithm for potential clinical applications (Kircher et al., 2021). Simultaneously, following the administration of a saline bolus, by calculating the SD of each pixel and plotting them at the relevant image position, a new type of EIT image presenting the exact position, into which the indicator is carried by the bloodstream and visualizing the temporal variation in perfusion distribution can be generated (Frerichs et al., 2002).

## Implementation of the Saline Bolus Method

The experimental and clinical studies using the saline bolus EIT method to visually assess lung perfusion are summarized in Tables 2–4 (Frerichs et al., 2002; Borges et al., 2012; Nguyen et al., 2013, 2015; Reinius et al., 2015; Hentze et al., 2018; Bluth et al., 2019; Grassi et al., 2020; He et al., 2020a,b,c, 2021b; Mauri et al., 2020; Safaee Fakhr et al., 2020; Kircher et al., 2021; Spinelli et al., 2021; Yuan et al., 2021). The administration of saline boluses, data analyses, precision, and cutoff values differ among studies, and a consensus must be reached regarding the clinical application of the saline bolus EIT method.

**Table 2:** Summary on indicator-enhanced EIT in 12 experimental studies.

**Researchers**	**Main research contents**	Subjects^*^	**Contrast agent**	**Apneic window**	**Data evaluation and main parameters**	**Comparison with validated imaging modalities**	**Conclusion**
Frerichs et al. (2002)	Contrast EIT imaging of regional pulmonary perfusion	3 pigs (20-kg mean body weight)	NaCl 5.85%, 10 ml	An end-expiratory pause of 2 min	Plot a local time- impedance change curve based on first-pass kinetics and obtain the peak impedance change	The **EBCT** scans showing the arrival of the radiographic material provide a visual reference for the EIT measurements.	EIT imaging of lung perfusion is feasible when an electrical impedance contrast agent is used.
Borges et al. (2012)	Regional pulmonary perfusion measured by EIT in artificially induced atelectatic model	6 piglets (2–3 years old weighing 28.4 ± 2.6 kg)	NaCl 20%, 5 ml	An expiratory pause of 2 min	Utilize the **maximal slope method** to calculate relative regional perfusion after extracting the net contrast curve within each pixel.	EIT showed good agreement with **SPECT** (mean difference was 0.6%, with a SD of 2.9%).	Indicator-enhanced EIT can effectively evaluate regional lung perfusion in healthy and impaired conditions.
Nguyen et al. (2013)	To explore the conductivity changes of four different impedance contrast boluses	a male Merino–cross weighing 64 kg	NaCl 3%, 60 ml (37°C); 5% glucose solution, 60 ml (21°C C); NaCl 20%, 10 ml (37°C); NaCl 3%, 60 ml (21°C).	~40 s	Calculate mean and standard deviation of the maximum relative conductivity change for each ROI induced by the indicator injection.	–	3% NaCl can produce comparable conductivity to 20% NaCl for pulmonary perfusion measurements.
Reinius et al. (2015)	To determine the online physiological effects of OLV/capnothorax	5 piglets (weighing 25–30 kg)	NaCl 20%, 5 ml	An expiratory pause of 20 s	Perfusion maps were pixel-wise calculated based on the impedance changes in response to hypertonic saline injection as described by Borges et al.	–	EIT can monitor real-time dynamic changes in pulmonary ventilation and perfusion distributions during sequential OLV/capnothorax at two different PEEP levels (5 and 10 cm H_2_O).
Nguyen et al. (2015)	To determine pulmonary perfusion defect caused by an artificially induced pulmonary embolism using contrast EIT	8 adult male Merino Cross (weighing 78 ± 7.8 kg)	NaCl 20%, 10 ml; NaCl 3%, 60 ml	40–45 s	Compute a right lung to left lung perfusion and estimate regional perfusion via calculating area under the contrast dilution curve.	–	EIT can reliably detect the difference between normal and embolized lung regions with unilateral perfusion defect as small as 8% of the lung using bolus of hypertonic saline 3%.
Hentze et al. (2018)	To assess the feasibility of obtaining regional ventilation-perfusion ratio (V/Q) by EIT	4 pigs (weighing 29–33 kg)	NaCl 10%, 10 ml	An expiratory pause of 1 min	Regional lung perfusion was extracted using a model-based approach based on semi-negative matrix factorization (Semi-NMF) and a gamma-variate model.	In dorsal-ventral direction, a strong agreement was detected between EIT and **SPECT** for both PEEP 0 (CoV = 11%) and 15 cm H_2_O (CoV = 13%). In right-left direction, correlations are strong (CoV = 18%) at PEEP 15 and moderate at 0 cm H_2_O (CoV = 30%).	The model-based approach is a powerful tool to extract perfusion from the indictor-based signal. Monitoring ventilation and perfusion by EIT is feasible.
Bluth et al. (2019)	Detection of acute changes in relative lung perfusion by contrast EIT imaging under different conditions of ventilation/perfusion matching	13 pigs (weighing 50–66 kg)	NaCl 3%, 10 ml; NaCl 10%, 10 ml; NaCl 5%, 10 ml	Not mentioned	Normalize the EIT signal to the overall detected signal and compute relative regional perfusion for each ROI.	Differences between regional perfusion measured by EIT and **PET** were relatively small (95% of values differed by <8.7, 8.9, and 9.5% for saline 10, 5, and 3%, respectively).	The agreement between EIT and PET for measuring and tracking changes of relative lung perfusion is satisfactory for clinical application.
Kircher et al. (2021)	To estimate regional pulmonary perfusion using contrast EIT under the conditions of acute lung injury	8 pigs (weighing 33.1 ± 2.2 kg)	NaCl 10% (0.75 ml/kg)	An end-expiratory pause of 35 s	A linearized and normalized time difference (LNTD) reconstruction algorithm was implemented to calculate the relative spatial distribution of pulmonary blood flow.	Strong correlation was found in dorsoventral (*r* = 0.92) and in right-to-left directions (*r* = 0.85) with good limits of agreement of 8.74% in eight lung segments between perfusion distribution detected by EIT and iodine contrast-enhanced **MDCT**.	Estimating regional perfusion by contrast EIT is feasible in experimental regional sublobar ARDS induced by saline lavage or endotoxin instillation.

* *All the subjects were anesthetized, paralyzed, and mechanically ventilated in a supine position*.

*EIT, electrical impedance tomography; EBCT, electron beam computed tomography; SPECT, single-photon emission computed tomography; PET, positron emission tomography; MDCT, multidetector computed tomography; ROI, region of interest; CoV, coefficient of variation*.

**Table 3:** Unmatched ventilation and perfusion measured by EIT in ICUs.

	**Researchers**	**Main research contents**	**Subjects**	**Contrast agent**	**Apneic window**	**Data evaluation**	**Conclusion**
Case reports	He et al. (2020a)	A clinical report using EIT and saline bolus to detect acute PE	A 47-year-old woman with multiple embolisms in right pulmonary artery branches suggested by CT	NaCl 10%, 10 ml	~8 s	EIT imaging presented normal ventilation distribution with massive perfusion defect in the affected right lung, leading to a ventilation-perfusion mismatch.	EIT might be helpful in monitoring patients with suspected PE at bedside via combining regional ventilation and perfusion information.
	Grassi et al. (2020)	An attempt of functional ventilation/perfusion imaging in a patient with acute PE	A 57-year-old woman with the left pulmonary artery embolism confirmed by CT.	Saline solution	Not mentioned	EIT-based functional perfusion and ventilation mapping of each ROI was used to generate ventilation/perfusion ratio (V/Q).	Bedside ventilation/perfusion images can assist to discriminate abnormal ventilation and perfusion situation during acute PE.
	Yuan et al. (2021)	An application of bedside EIT to dynamically assess regional pulmonary perfusion defect in a patient with acute massive PE	A 68-year-old man suffering acute massive PE confirmed by CTPA.	NaCl 10%, 10 ml	Not mentioned	Normal ventilation distribution with massive defects in regional perfusion led to ventilation–perfusion mismatch in both lungs.	EIT might have the potential to assess and monitor regional perfusion for rapid diagnosis of fatal PE in clinical practice.
	Safaee Fakhr et al. (2020)	The use of EIT to diagnose a significant lung perfusion defect and to assess the response to anticoagulative therapy in a patient with COVID-19 pneumonia	A 66-year-old man diagnosed COVID-19 pneumonia with progressive PE confirmed by CTPA.	Not mentioned	Not mentioned	EIT showed homogenous ventilation and imbalanced perfusion distribution with dead space% estimated at 66%, which was consistent with a major perfusion impairment caused by PE.	Bedside EIT may be helpful to identify perfusion impairment and assess pulmonary perfusion responded to therapeutic management over time.
Clinical trials	He et al. (2020b)	To investigate the association between PEEP-induced lung overdistension/recruitment and V-Q match by EIT.	30 adult mechanically ventilated patients: 18/30 with ARDS and 12/30 with high risk for ARDS.	NaCl 10%, 10 ml	An end-expiratory pause (>8 s)	The functional perfusion map was calculated as the slope of regional impedance–time curves. Dead Space%, Shunt%, and V-Q match% were calculated based on EIT perfusion and ventilation images.	Changes of ventilation–perfusion matching were associated with regional overdistention/recruitment induced by PEEP in patients with ARDS.
	He et al. (2020c)	To confirm the feasibility of bedside detection of acute PE using EIT with saline bolus injection.	68 patients (ventilated or conscious) with ARF: 11/68 with PE (10 confirmed by CTPA) and 57 without PE.	NaCl 10%, 10 ml	An end-expiratory pause (≥8 s)	Same as above	EIT-based regional ventilation and perfusion measurements were able to discriminate patients with acute PE from other patients with ARF.
	He et al. (2021b)	To validate whether regional ventilation and perfusion data measured by EIT with saline bolus could discriminate three broad acute respiratory failure (ARF) etiologies.	108 ICU patients (93 with ARF and 15 without as a control).	NaCl 10%, 10 ml	An end-expiratory pause (>8 s)	Same as above	It was feasible to characterize three broad etiologies of ARF with EIT-based regional ventilation and perfusion.
	Mauri et al. (2020)	To describe specific pathophysiological characteristics of C-ARDS using EIT with saline bolus injection.	10 intubated patients (54–64 years old) with C-ARDS	NaCl 5%, 10 ml	An end-inspiratory pause of 20 s	Pixel-level relative regional pulmonary perfusion and ventilation signals were normalized to compute Dead Space%, Shunt%, V/Q mismatch, and the dead space to shunt ratio.	Elevated dead space% might be a specific pathophysiological trait in patients with C-ARDS.
	Perier et al. (2020)	To describe the physiological effects of PEEP and prone position on pulmonary perfusion by EIT in patients with C-ARDS.	9 deeply sedated and paralyzed patients with C-ARDS	NaCl 7.5%, 10 ml	An expiratory pause	Calculate impedance change caused by ventilation (ΔZV) and perfusion (ΔZQ), cardiac output, alveolar ventilation volume and V/Q ratio at pixel level in dependent and non-dependent area of the lung.	Prone positioning and increased PEEP resulted in better V/Q match in patients with C-ARDS.
	Spinelli et al. (2021)	To quantify V/Q mismatch by EIT investigating their association with mortality in patients with ARDS and to explore the effects of PEEP on V/Q mismatch in ARDS of different severity.	50 mechanical ventilated, deeply sedated and paralyzed patients with ARDS of different severity.	NaCl 5%, 10 ml	An end-inspiratory pause of 20 s	Pixel-level relative perfusion and ventilation signals were calculated to classify non-perfused and non-ventilated regions, yielding only perfused units%, only ventilated units%, unmatched units% and V/Q ratio in each region.	EIT allowed bedside assessment of V/Q mismatch in patients with ARDS, which could identify patients at higher risk of death and guide personalized treatment.

*PE, pulmonary embolism; ARDS, acute respiratory distress syndrome; ARF, acute respiratory failure; CTPA, computed tomographic pulmonary angiography; C-ARDS, ARDS resulted from COVID-19; COVID-19, coronavirus disease 2019; PEEP, positive end-expiratory pressure*.

**Table 4:** Summary on indicator-enhanced EIT in animal studies and clinical studies.

**Research content**	**Animal study**	**Clinical study**
PE (using regional lung perfusion/dead space to diagnosis PE)	2 studies: (Frerichs et al., 2002; Nguyen et al., 2015)	5 studies (4 case reports, 1 observational study): (Grassi et al., 2020; He et al., 2020a,c; Safaee Fakhr et al., 2020; Yuan et al., 2021)
ARDS (lung perfusion of COVID-19, response of PEEP and Prone position, prognosis)	3 studies: PEEP selection: (Hentze et al., 2018; Bluth et al., 2019; Different ARDS models: Kircher et al., 2021)	4 observational studies: (He et al., 2020b; Mauri et al., 2020; Perier et al., 2020; Spinelli et al., 2021)
Comparison with PET/SPECT/MDCT	5 studies: (Frerichs et al., 2002; Borges et al., 2012; Hentze et al., 2018; Bluth et al., 2019; Kircher et al., 2021)	–
Effectiveness of Contrast Agents	3 studies: (Nguyen et al., 2013, 2015; Bluth et al., 2019)	–

*PE, pulmonary embolism; ARDS, acute respiratory distress syndrome*.

### Breath Holding

With the aim of reducing the influence of tidal impedance, breath-holding is an important part of saline bolus implementation. It remains controversial how the breath should be held and how it should be implemented.

#### How Long Does the Breath-Holding Maneuver Last?

The mean lung transit time, which is defined as the time required for blood flow transfer from the right ventricle to the left atrium, should be taken into consideration regards to the length of the breath-hold. However, the apnea time does not need to be as long as the pulmonary transit time (PTT) for lung perfusion (He et al., 2021a). Recent experimental explorations suggested that it took ~3–5 s from the injection for the saline to enter the lungs and ultimately concentrate (Hentze et al., 2018). An 8- to 20-s breath-hold maneuver was used in different experimental and clinical studies (Table 2). Breath-holding for a longer duration might demand neuromuscular blocking agents. In contrast, breath-holding for a shorter time might be more practicable at the bedside. Our experience showed that deep sedation (Richmond Agitation Sedation Scale at—4) was enough to obtain a breath hold of 8 s. Moreover, it should be considered that some conditions, such as extremely low/high cardiac outputs, have apparent abnormal pulmonary transit times. Further study is required to optimize the breath-holding time and ensure the effective acquisition of the entire impedance curve.

#### How Should Breath-Holding Be Performed?

Both end-expiratory and end-inspiratory holds were used during the saline bolus implementation for lung perfusion measurement. The end-expiratory hold had the following potential benefits: (1). little impact on venous return and circulation; (2). a distinct impedance change might be more easily caused by a saline bolus at end-expiration with a lower global impedance baseline. On the other hand, end-inspiratory holding with maximal inflation of the lungs can suppress respiratory drive and dilute carbon dioxide (CO_2_) levels, and it is considered to be more readily implemented. However, the higher airway pressure at end-inspiration may cause an impairment of global circulation and lung perfusion (He et al., 2021a). Further exploration is necessary to compare the clinical applicability of end-inspiration and end-expiration occlusion methods.

### Saline Bolus Injection

Briefly, breath-holding was required during the saline bolus injection. If spontaneous breathing was present during the saline bolus administration, the impedance–time curve would be unfit for assessing lung perfusion. It is worth noting that the saline bolus must be injected manually as soon as possible (<2 s) through the central venous catheter (He H. W. et al., 2021).

Due to its high conductivity, hypertonic saline was used as an EIT contrast agent. The concentrations (from 3 to 20%) and volume (from 5 to 60 ml) of saline solution varied among different studies (shown in Tables 2, 3). Technically, a higher concentration of saline solution could improve the signal-to-noise ratio of EIT imaging but could introduce safety risks regarding potential pulmonary fluid transfer and a faster variation in hemodynamic parameters (Hellige et al., 2012). There has been no consensus on the frequency of the saline bolus injection over 1 day. However, caution should be paid to excessive saline infusion that could disturb electrolyte balance.

### EIT Data Analysis

**Figure 1:**
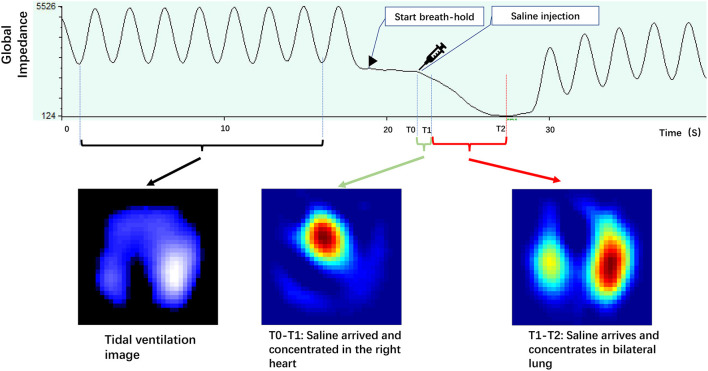
Illustrates impedance–time curves and corresponding ventilation/perfusion images during the saline bolus. The tidal impedance variation during normal tidal breathing before apnea was used to calculate the tidal ventilation image. The impedance–time curve caused by saline bolus during the apnea period was used for the perfusion image (adapted from our recent paper).

Functional ventilation maps were derived by averaging the tidal impedance variation images. Functional perfusion maps were calculated as the slope of the regional impedance–time curves after the saline bolus injection. Figure 1 illustrates the impedance–time curves and the corresponding ventilation/perfusion images following the injection of a saline bolus.

The perfusion value of pixel *i P*_*i*_ in the perfusion image was equal to –*a*_*i*_. Furthermore, the ventilated and perfused regions were defined as follows: region *k* was ventilated if


(1) $$\begin{array}{r} V_{k}>20\%\times \max\left(V_{K}\right)\begin{array}{cc} , & K\in \left[1,1024\right] \end{array} \end{array}$$

and, similarly, region g was perfused if


(2) $$\begin{array}{r} P_{g}>20\%\times \max\left(P_{G}\right)\begin{array}{cc} , & G\in \left[1,1024\right] \end{array} \end{array}$$

Moreover, regional ventilation/perfusion ratio (V/Q) matching could be obtained based on the combination of the ventilation and perfusion distribution images from EIT (He et al., 2020b,c). Three regions were identified based on the ventilation/perfusion patterns: regions that were only ventilated (RVs), regions that were only perfused (RPs), and regions that were both ventilated and perfused (RV + Ps). The following V/Q matching from the EIT-derived parameters was obtained:


(3) $$\begin{array}{r} DeadSpace_{\%}=R_{V}/\left(R_{V}+R_{P}+R_{V+P}\right)\times 100\% \end{array}$$

(4) $$\begin{array}{r} Shunt_{\%}=R_{P}/\left(R_{V}+R_{P}+R_{V+P}\right)\times 100\% \end{array}$$

(5) $$\begin{array}{r} VQMatch_{\%}=R_{V+P}/\left(R_{V}+R_{P}+R_{V+P}\right)\times 100\% \end{array}$$

Mauri and Spinelli et al. defined non-ventilated or non-perfused pixels when the V_pixel_ or Q_pixel_ were less than or equal to 10% of the highest pixel-level impedance change in a similar method (Mauri et al., 2020). Subsequently, the percentage of the pixels with a V/Q mismatch was calculated to investigate the relationship between the mismatches and the prognoses of ARDS (Spinelli et al., 2021).

Recently, Perier et al. developed an analytical method for assessing lung perfusion and V/Q matching with EIT (Perier et al., 2020), including methods for determining the impedance changes caused by ventilation (ΔZ_V_) and perfusion (ΔZ_Q_), cardiac output, and alveolar ventilation volume. The authors assumed a 30% fixed anatomical dead space; the V/Q matching was calculated pixel by pixel, and then relevant parameters were defined:


$$\begin{array}{r} \frac{V_{\left(Pixel\right)}}{Q_{\left(Pixel\right)}}=\left[\frac{\Delta Z_{V\left(Pixel\right)}}{\Delta Z_{V\left(Total\right)}}\times \left(V_{T}\times respiratory\;rate\times 0.7\right)\right]/ \end{array}$$

(6) $$\begin{array}{r} \left[\frac{\Delta Z_{Q\left(Pixel\right)}}{\Delta Z_{Q\left(Total\right)}}\times CO\right] \end{array}$$

$$\begin{array}{r} \text{Shunt}\sim \text{severe if V}/\text{Q }<\;0.1;\text{ moderate if V}/\text{Q between} \end{array}$$

(7) $$\begin{array}{r} \;0.1\;and\;0.5 \end{array}$$

$$\begin{array}{r} \text{Dead Space}\sim \text{severe if V}/\text{Q }>10;\text{ moderate if V}/\text{Q between} \end{array}$$

(8) $$\begin{array}{r} 2\text{ and }10 \end{array}$$

Moreover, Nguyen et al. compared the different parameters of the averaged contrast dilution curve, including the peak value, maximum uptake, maximum washout, and area under the curve to estimate the relative distribution of pulmonary perfusion in an animal model of pulmonary embolism-like events (Nguyen et al., 2015). The authors found that the right lung-to-left lung perfusion ratios of the area under the curve and the peak value of the averaged contrast dilution curve are the most promising and reliable parameters in assessing pulmonary embolism (PE). Variation was found among different impedance–time curve analysis methods in two patients (He et al., 2021a), and further study is required to optimize the analysis methods.

## Applications of Contrast EIT in Animal and Clinical Studies

The availability of contrast EIT presented in multiple experimental and clinical studies allowed the instant measurement of lung regional perfusion changes under various pathological conditions, such as PE, atelectasis, and ARDS (Frerichs et al., 2002; Borges et al., 2012; He et al., 2020a,c; Mauri et al., 2020; Kircher et al., 2021). A remarkable agreement between EIT and SPECT further proved the correctness and practicability of this emerging perfusion imaging method.

### Animal Studies

We summarized eight animal studies on conductivity contrast bolus EIT, four of which compared EIT and SPECT perfusion results and presented significant consistency (Table 2).

In a regional lung artery occlusion model, Frerichs et al. were the first to demonstrate the feasibility of EIT imaging in detecting pulmonary perfusion impairments when a conductive contrast agent was used (Frerichs et al., 2002). A strong correlation was found in the paired measurements of the perfusion distribution by EIT and SPECT, with a mean difference of 0.6% and an SD of 2.9% (Borges et al., 2012). In addition, the redistributive pulmonary perfusion, as estimated by EIT, with a distinct decrease in perfusion within the injured lung compartment between the injured and healthy regions, was validated by a dynamic multidetector computed tomography (MDCT) in a study of animal models with induced regional sublobar ARDS with reasonable limits of agreement value of 8.74% (Kircher et al., 2021).

### Clinical Studies

The following section summarizes nine clinical studies on contrast EIT, including four case reports and five clinical trials (Table 3).

It was demonstrated that EIT perfusion imaging is accurate and effective in diagnosing and monitoring the therapeutic response of PE at the bedside. The normal ventilation distribution with massive perfusion defects in the affected regions as determined by EIT imaging showed strong diagnostic efficiency in studies comprising subjects suffering multiple embolisms confirmed by CT pulmonary angiography (Grassi et al., 2020; He et al., 2020a,c; Safaee Fakhr et al., 2020; Yuan et al., 2021). He et al. found that patients with PE had significantly higher dead space% and lower V/Q match% than patients without PE in a prospective observational study (He et al., 2020c). A cutoff value of 30.37% for dead space% resulted in a sensitivity of 90.9% and a specificity of 98.6% for PE diagnoses (He et al., 2020c). Moreover, the same team utilized saline bolus EIT to assess the relationship of ventilation–perfusion matching with regional overdistention and recruitment induced by PEEP in patients with ARDS (He et al., 2020b).

Marui et al. found that EIT could detect imbalanced ventilation/perfusion matches based on ventilated areas with impaired perfusion in ARDS resulting from COVID-19 (C-ARDS) and predict the therapeutic outcomes of ARDS patients when considering a variety of EIT-based information (Mauri et al., 2020; Perier et al., 2020; Spinelli et al., 2021). Perier et al., using EIT, confirmed that prone positioning and increased PEEP resulted in better V/Q matches in patients with C-ARDS (Perier et al., 2020).

## Prospects and Limitations

Saline bolus EIT can determine differences in regional perfusion resulting from perfusion distributions reflecting the diseases, therapeutic maneuvers (e.g., administration of vasoactive agents, and mechanical ventilation), and ventilation/perfusion maps of multiple pathophysiological phenomena. Therefore, it is possible for the contrast EIT method to have clinical applications in the future. First, saline bolus-based EIT might have the potential to identify various etiologies of acute respiratory failure (ARF). Recently, He et al. demonstrated that contrast EIT-derived regional ventilation and perfusion measurements were able to characterize three broad ARF etiologies, including pulmonary embolism-related disease (PED), diffuse lung involvement disease (DLD), and focal lung involvement disease (FLD) (He et al., 2021b). Second, for mechanically ventilated patients, increasing clinical evidence has shown that perfusion EIT could provide bedside guidance for tidal setting, PEEP titration, and prone ventilation and help physicians evaluate the prognosis of patients with ARDS. Third, this saline bolus-based EIT method featured with convenience and promptness might be able to satisfy the clinical use for monitoring therapeutic effects for severe patients, for example, to assess the effects of inhaled medicine to PH and the therapeutic effect of anticoagulation/thrombolytic therapy on PE.

For the extensive application of the saline bolus EIT lung perfusion method in the ICU, more clinical researches and experimental studies are required to confirm the accuracy and validity of saline contrast EIT. Importantly, there is a strong need for a consensus on the cutoff value to determine pulmonary perfusion parameters, like the one for ventilated/perfused units in assessing V/Q mismatch (He et al., 2020b,c; Mauri et al., 2020; Spinelli et al., 2021). Although there has been little evidence showing the feasibility of EIT perfusion imaging in patients with spontaneous breathing, there is a great chance that breath-holding maneuvers adjustive to awake patients like inspiratory holds using typical entry standards are next in line. Furthermore, to achieve the continuous monitoring of pulmonary blood flow, it is foreseeable that the combination of contrast EIT with pulsatility-based EIT for dynamic assessments will be explored in the future.

However, the EIT analysis has drawbacks in providing detailed morphological information to accurately identify the vertical location of perfusion deficits attributed to restrained spatial resolution. Hence, the reconstruction algorithm warrants further optimization to provide robust evaluations in clinical applications. Additionally, using a single-ring external electrode, the EIT method is only allowed to monitor one cylindrical lung part at one time, instead of providing an overall lung image like CT and MRI. Moreover, auxiliary evaluations or data processing procedures to address the conductivity variations are necessary to accommodate background conductivity changes when a conductive contrast agent is used (Bluth et al., 2019). Finally, despite the validation in intubated subjects, the feasibility of perfusion EIT in spontaneous respiratory patients is yet to be fully understood. Since the effectiveness of the breath-holding maneuver may vary due to the individual variations in conscious patients, contrast EIT focusing on applications in non-mechanically ventilated patients demands future discussion before being used in clinical practice.

## Conclusions

Using a hypertonic saline solution based on first-pass kinetics, contrast EIT imaging generating dilution impedance waveforms is reliable for determining lung perfusion changes reflecting atelectasis, spatial heterogeneity, and artery embolism in a regional manner. With the ability to monitor regional lung perfusion and produce V/Q mapping, saline bolus EIT is promising and meaningful for patients in ICUs. Further study is required to validate the impact of the described saline bolus EIT method on decision-making, therapeutic management, and outcomes in critically ill patients.

## Author Contributions

MX, HH, and YL contributed to conception and design of the review. MX and HH searched and organized the database. MX wrote the first draft of the manuscript. HH and YL wrote and refined several important sections of the manuscript. All authors contributed to manuscript revision, read, and approved the submitted version.

## Funding

This study was supported by the CAMS Innovation Fund for Medical Sciences (No. CAMS Innovation Fund for Medical Sciences (No. 2020-I2M-C&T-B-042), Capital's Funds for Health Improvement and Research (No. 2020-2-40111), Excellence Program of Beijing Clinical Key Specialty for Critical Care Medicine (2020), and Beijing Municipal Science and Technology Commission (Grant No. Z201100005520051).

## Conflict of Interest

The authors declare that the research was conducted in the absence of any commercial or financial relationships that could be construed as a potential conflict of interest.

## Publisher's Note

All claims expressed in this article are solely those of the authors and do not necessarily represent those of their affiliated organizations, or those of the publisher, the editors and the reviewers. Any product that may be evaluated in this article, or claim that may be made by its manufacturer, is not guaranteed or endorsed by the publisher.
